# Supplementary material for: Indirect Effects of the COVID-19 Pandemic on Routine Childhood Vaccination in Low-Income Countries: A Systematic Review to Set the Scope for Future Pandemics
Source: Microorganisms. 2024 Mar 13;12(3):573. doi: 10.3390/microorganisms12030573 (PMC10972497; doi:10.3390/microorganisms12030573)
Supplement: Supplementary file 1 [file microorganisms-12-00573-s001.zip › microorganisms-2892851-supplementary.pdf]

Table S1. Search strategy executed February 9, 2023 on Ovid MEDLINE(R) and Epub Ahead of Print, In-Process, In-Data-Review and Other Non-Indexed Citations and Daily (1946 to February 8, 2023).

Number	Items	Hits
1	exp Immunization	205344
2	(immuniz$ or vaccinat$ or vaccine$).mp	549975
3	1 or 2	567991
4	(low$ income$ adj1 (countr$ or econom$ or nation$)).mp	10294
5	(afghan$ or burkina$ faso$ or burundi$ or chad$1 or congo$ or eritrea$ or ethiop$ or gambia$ or guinea$ or north$ korea$ or liberia$ or madagascar$ or malawi$ or mali$1 or mozambiqu$ or niger$1 or rwanda$ or sierra$ leone$ or somolia$ or sudan$ or syria$ or togo$1 or uganda$ or yemen$ or zambia$).mp	354367
6	(korea$ adj2 democra$ peopl$ repub$).mp	377
7	central$ afric$ repub$.mp	1359
8	or/4-7	363421
9	3 and 8	21104
10	SARS-CoV-2	147281
11	COVID-19	211672
12	covid$.mp	325261
13	(2019-ncov$ or 2019ncov$ or ncov2019$ or ncov-2019$).mp	2866
14	((wuhan$ or hubei$) and corona$).mp	6849
15	(sars-cov-2$ or sars-cov2$ or sarscov2$ or sars$ corona$ 2$).mp	193636
16	(("2019" or "2020" or "2021" or "2022" or "2023") and corona$).mp	91882
17	or/10-16	343648
18	9 and 17	886
19	(pediatric$ or paediatric$ or child$ or infan$ or neonat$ or neo-nat$ or newborn$ or new$ born$ or adolescen$ or teen$ or youth$).ti,hw,kf,jw	4466230
20	((routin$ or schedule$ or catchup$ or catch$ up$) adj1 (vaccin$ or immuniz$)).ti,hw,kf.	12393
21	((well$ child$ or well$ bab$ or well$ infan$ or well$ check$ or well$ visit$) adj4 (vaccin$ or immuniz$)).mp	200
22	or/19-21	4470559
23	18 and 22	120

24	..l/ 23 lg=en	118
25	..l/ 24 yr=2020-current	118
26	remove duplicates from 25	118

Abbreviations: exp = explode, mp = multi-purpose, $ = truncation to retrieve alternative endings, adj = adjacency, ti = title, hw = heading word, kf = keyword heading word, jw = journal word.

Table S2. Risk of bias of included studies using ROBINS-I tool.

Citation	Bias due to confounding	Bias in selection of participants into the study	Bias in measurement of exposures or outcomes	Bias due to missing data	Bias in selection of studies or reported outcomes
Abid 2022	Low	Low	Low	Moderate	Low
Arsenault 2022	Low	Low	Low	Moderate	Low
Buonsenso 2020	Low	Moderate	Low	Moderate	Low
Burt 2021	Low	Low	Low	Moderate	Low
Connolly 2022	Low	Low	Low	Moderate	Low
Das Neves Martins Pires 2021	Low	Low	Moderate	Moderate	Low
Gebreegziabher 2022	Low	Low	Low	Moderate	Low
Kassie 2021	Low	Low	Low	Moderate	Low
Kotiso 2022	Low	Low	Low	Moderate	Moderate
Masresha 2020	Low	Low	Moderate	Moderate	Low
Osei 2022	Moderate	Low	Low	Low	Low
Shapira 2021	Low	Low	Low	Moderate	Low
Wanyana 2021	Low	Low	Low	Moderate	Low
